# CNS active target (CAT) for missing mass spectroscopy with intense beams

**DOI:** 10.1007/s10967-015-4130-5

**Published:** 2015-05-08

**Authors:** S. Ota, H. Tokieda, C. S. Lee, Y. N. Watanabe

**Affiliations:** Center for Nuclear Study, The University of Tokyo, 2-1 Hirosawa, Wako, Saitama 351-0198 Japan; RIKEN Nishina Center for Accelerator-Based Science, 2-1 Hirosawa, Wako, Saitama 351-0198 Japan; Department of Physics, The University of Tokyo, 7-3-1 Hongo, Bunkyo, Tokyo 113-8654 Japan

**Keywords:** Deuterium, Gas target, Active target, THGEM, Time projection chamber

## Abstract

A new gaseous active target based on a time projection chamber, named CAT, is introduced. The remarkable feature is a dual gain THGEM to decrease the effective gain for the beam particles while keeping a high enough effective gain for the recoil particles. The measured effective gain of low gain region was a factor of one hundred smaller than that of high gain region. This technique provides a wide dynamic range in order to detect both the beam and recoil particles at the same time even with a very high intensity beam of more than 10^5^ Hz.

## Introduction

**Fig. 1:**
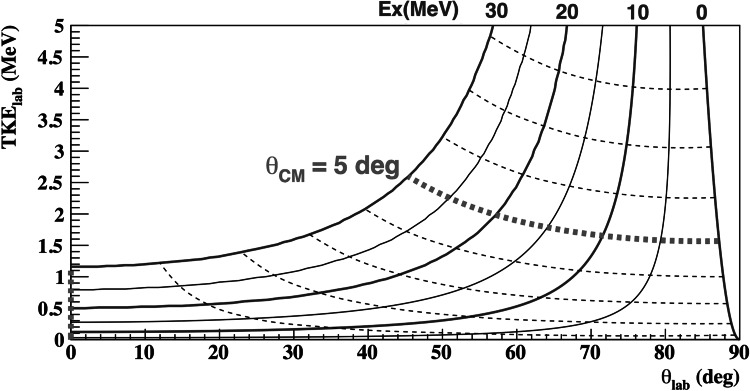
The kinematical correlation between the recoil angle and the total kinetic energy in deuteron scattering off ^132^Xe is displayed. The *horizontal* and *vertical* *axes* show the recoil angle and the total kinetic energy of the recoil deuteron in laboratory frame, respectively. The *solid curves* indicate the excitation energy of ^132^Xe in 5-MeV steps. The *dotted curves* indicate the scattering angle in the center-of-mass frame in one-degree steps

Deuteron-induced reactions such as (d,d′) and (d,2p) are useful spectroscopic tools to study the isoscalar-type response as well as the charge-exchange spin-flip response, respectively. Especially with these reactions, monopole components are of interest, which are relevant, for example, to investigate the equation of state of asymmetric nuclear matter, and the electron capture rate related to supernova explosions and supernova nucleosynthesis. Reactions which populate the monopole component have forward-peaked angular distributions in the center-of-mass frame, which is an important fact when designing an appropriate laboratory setup to perform the measurements of such reactions. Missing mass spectroscopy, in which the momentum of the probe particle is measured, is a promising tool because the measurement of the probe particle is independent from the decay properties of the excited nucleus. As a consequence, missing mass spectroscopy covers a wide range of excitation energy above the particle threshold, where the strong monopole component exists, on the same footing as below the threshold. In order to apply this method to unstable nuclei, a radioactive beam and inverse kinematics are required, and one needs to measure the target-like recoil particle. The kinetic energy of the recoil particle at forward angles is very small, as described presently. A gaseous active target based on a time projection chamber (TPC) enables us to measure such low-energy recoil particles, even if the recoil particle would not have enough energy to escape from a solid target of the same thickness as the active target. Figure 1 shows the kinematic correlation between the recoil angle and the total kinetic energy of recoil particle in laboratory frame in the ^132^Xe(d,d′) reaction in inverse kinematics at the incident energy of 100 MeV per nucleon. The solid and dotted curves show the excitation energy and the scattering angle in the center-of-mass frame, respectively. The TPC in the CAT is designed to have a sensitivity to recoil particles having 300-keV total kinetic energy, which corresponds to the scattering at around two degrees in the center-of-mass region for the excitation energy of 15 MeV, which is the typical energy of giant monopole resonance in the Xe-region of the chart of nuclides.

In an active target, the energy deposit is measured not only for recoil particles but also for beam particles. The energy deposit of the beam particle is much larger than that of recoil particles. In ^132^Xe(d,d′) inelastic scattering at 100 MeV per nucleon, for instance, the energy deposit of the beam particle in the active target is a factor of one hundred larger than that of the recoil particle. As the beam intensity is increased, a large number of electrons are produced by the beam particles, causing a huge variation of the applied voltage to the multiplication component of the active target and resultant gain variation. The large number of feedback ions from the multiplication component may distort the drift field. Furthermore, high-momentum electrons produced by the beam particles can obscure the true events. In order to cope with these effects arising from high-intensity, heavy-ion beam, various methods have been employed, such as a beam mask in MAYA [1] and a cathode shield surrounding the beam in TACTIC chamber [2]; these systems made it possible to perform experiments with beam intensities greater than 10^7^ Hz. In the CAT, a mesh grid was introduced to reduce the effective gain as described in Ref.  [3], and the gain was reduced by a factor of one hundred, which stabilized the applied voltage. However the charge resolution was worse than the goal of 10 %, since the number of primary electrons was reduced by a larger amount than expected, and the statistical error increased. In order to reduce the effective gain while retaining the charge resolution for beam particle, instead we decreased the effective gain along the beam path by separating the multiplication part into different regions for the beam and recoil particles. In this paper, we described the CNS Active Target system, and we report the measurements of the effective gain and resolution with dual gain THGEM using a high-intensity heavy-ion beam.

## CNS active target

**Fig. 2:**
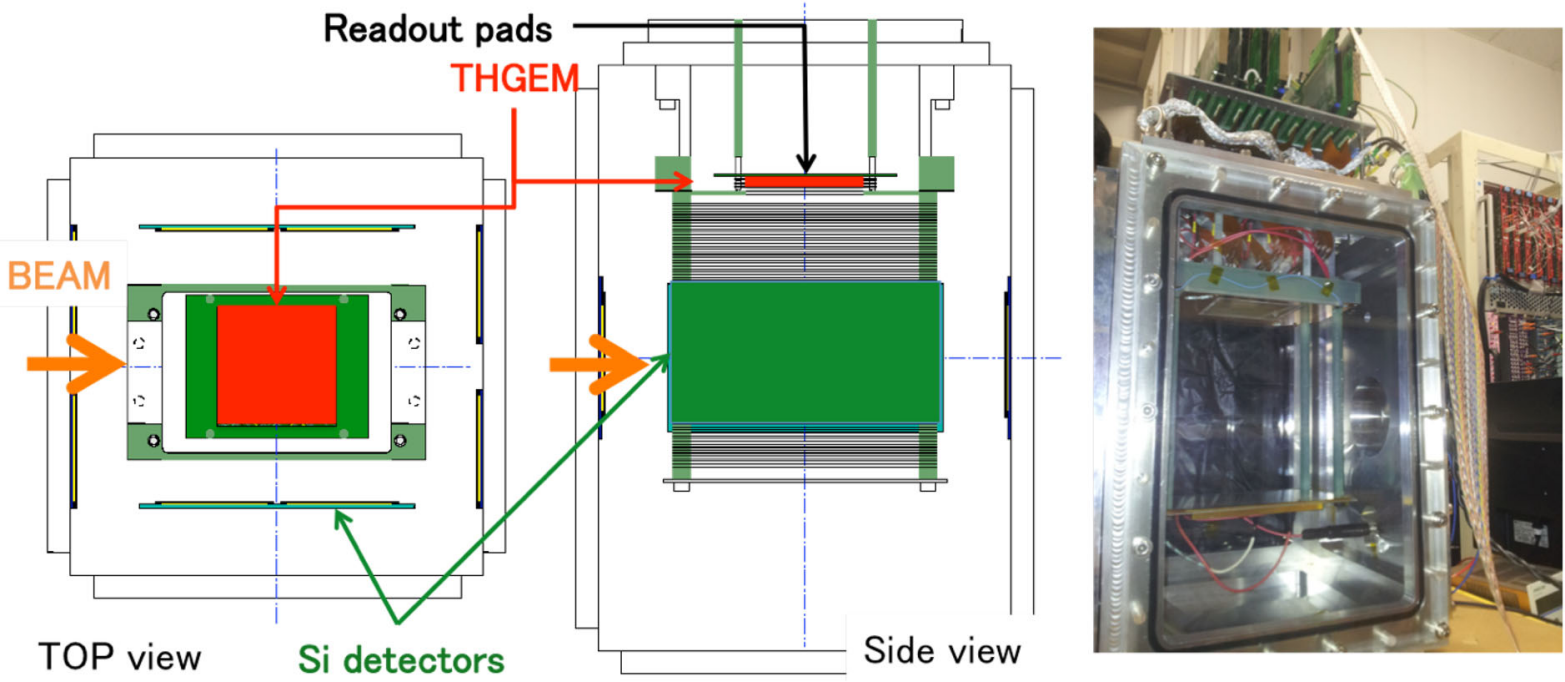
Schematic *top* and *side* views of the CAT are shown at the *left* and *center panels*, respectively. The CAT consists of a TPC and Si detector array surrounding the TPC field cage. The TPC has 10 × 10 × 25 cm^3^ active volume, and the typical field strength of the TPC drift region is 1 kV cm^−1^ atm^−1^. At a pressure of 0.4 atm, the typical voltage setting for the top plate is 14 kV. The Si detectors are 0.5-mm thick and have a sensitive area 9 × 9 cm^2^. The *right panel* shows a photograph of the CAT

The CNS active target (CAT) consists of a time projection chamber (TPC) and Si (or NaI) detector array, as shown in Fig. 2. The CAT is designed to cover the scattering angles from one degree [3, 4]. The photograph of the CAT is displayed in the right panel in Fig. 2. The Si detectors with dimension of 9 cm by 9 cm and 0.5-mm thickness surround the TPC. The TPC consists of a field cage, electron multipliers and readout pads. The typical electric field applied to the field cage is 1 kV cm^−1^ atm^−1^. Thick gas electron multipliers (THGEMs) [5, 6] with 10 × 10 cm^2^ active area are located at the center of the TPC. The thickness, the hole diameter, and pitch of the THGEM are 400, 300 and 700 μm, respectively. In order to detect the scattered target-like light particles, which require a gain of more than 5000, three layers of THGEMs are needed according to the effective gas gain reported in Ref. [7]. The distance between the THGEMs is 2 mm. Readout pads are of equilateral triangle shape and 120 such pads with 10-mm length sides and 280 pads with 5-mm length sides are located at the recoil and beam regions, respectively. There are 400 channels in total in the CAT system. A preamplifier (RPA-211 REPIC) followed by a buffer amplifier integrates charges collected at the readout pad. The output of the buffer amplifier is digitized by a V1740 module (CAEN) with a 50-MHz sampling rate. All the data in the V1740 module are transferred to a personal computer via A3818 PCI express card (CAEN). The data are reduced by software zero suppression. The V1740 module can provide trigger output by an internal threshold setting. The trigger is generated as the logical-OR of the trigger outputs and the other external trigger signals, which are received from the beam monitors, for instance. The maximum acceptable trigger rate is around 300 Hz.

## Dual gain THGEM and the measurements of the effective gain

**Fig. 3:**
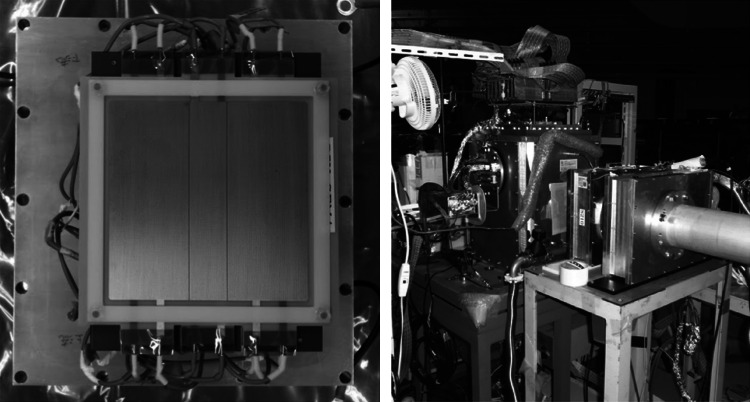
The *left panel* shows the photograph of the Dual Gain THGEM. In the *right panel*, the photograph of the experimental setup at HIMAC is displayed. The beam travels from right side to left side. The CAT is located at downstream of the tracking detector connected to the beam pipe

In the measurement of the (d,d′) and (d,2p) reactions, the luminosity should be as high as possible. Our goal is to achieve successful operation under high intensity beam injection such as 10^6^ Hz. In the case of the TPC, the main limitation will be the instability due to large current produced by multiplied electrons (and ions) and the pile up of the output signal. At a typical pulse width of 1 μs, the maximum available intensity will be 10^6^ Hz. With such a high intensity beam, the current produced by the multiplied electrons (or ions) becomes quite large. The energy deposit by a heavy-ion beam particle such as Xe at 100 MeV per nucleon is typically 120 keV mm^−1^, which corresponds to 50-fC charge along the 10-cm flight path in the active region. If the effective gain of the multiplication part is 10^4^, the current induced at the last stage of the multiplication part becomes 500 μA. Some fraction of this current goes to the THGEM. This current causes an instability of voltage applied to the THGEM, results in a variation of the gain. To avoid this instability, we employed Dual Gain THGEM (DGTHGEM). The left panel in Fig. 3 shows a photograph of the DGTHGEM. The electrodes at the top and bottom sides of the DGTHGEM are divided into three regions. The width of the center region is 2 cm to cover the envelope of the beam. The width of the side region is 4 cm on both sides. The voltage settings for the two side regions are common and the voltage setting for the central region differs from that for the side regions in order to decrease the effective gain. Since the central region cannot also be sensitive to the recoil particles, the angular coverage at the forward angles below 1° in center of mass frame is not possible in such a measurement with DGTHGEMs.

The test experiment to measure the effective gain of the low gain region of DGTHGEM was performed at Heavy Ion Medical Accelerator in Chiba (HIMAC) in the National Institute of Radiological Sciences (HIMAC collaborative research No. 12H307). The right panel in Fig. 3 shows the photograph of the experimental setup. The primary beam of ^132^Xe with an energy of 100 MeV per nucleon was injected into the CAT. The injection intensity ranged from 10^3^ to 10^6^ particles per pulse. The CAT was operated with 0.4-atm deuterium gas and three layers of DGTHGEMs. The field strength of the drift region was about 1 kV cm^−1^ atm^−1^. The voltage settings of the side regions of the DGTHGEMs were set so as to achieve the effective gain higher than 5000.

**Fig. 4:**
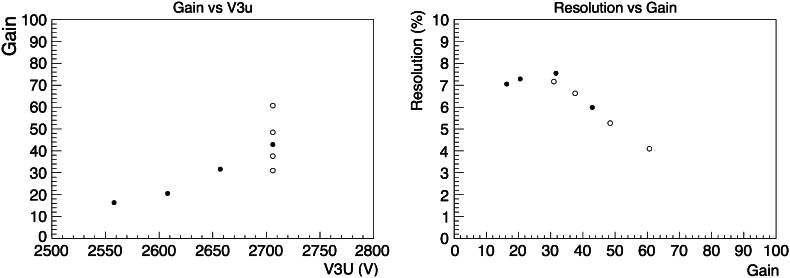
The effective gain in the low gain region along the beam path is shown as a function of the voltage supplied to the top of DGTHGEMs in the *left panel*. The *vertical axis* shows the effective gain and the *horizontal axis* shows the voltage value of the top of DGTHGEMs. The charge resolutions in sigma as a function of the effective gain are shown in the *right panel*. The *vertical* and *horizontal axes* show the resolution and effective gain, respectively. In the both panels, the *solid circles* indicate that the ratio among the applied voltages is fixed, and the *open circles* indicate the voltages to the third DGTHGEM are changed

The left panel of Fig. 4 shows the measured effective gain along the beam path as a function of the voltage applied to the electrode nearest to the field cage. Solid circles show the data where the ratio among the bias voltages of the three layers of DGTHGEMs is constant. The bias voltages at the data point of 2657 V are −2657, −2087, −1746, −1340, −1060, −370 V. Open circles show the data where constant voltages were applied to the electrodes other than the one nearest to the readout pads. The voltage to that electrode was varied in 10-V steps. The effective gain is the ratio of the sum of the collected charge along the 10-cm beam path (which corresponds to 40 pads in total in two neighboring rows) to the charge produced assuming an energy deposit of 12 MeV in an energy-loss calculation. The achieved gain was less than one hundred for all the settings. The resolution as a function of the gain is shown in the right panel in Fig. 4. The resolution is obtained as the standard deviation of the charge distribution, since the energy loss straggling is 0.5 % according to the calculation.

The measured resolutions are better than 10 % and the resolution becomes better as the gain increases. The absolute value of the resolution is worse than the calculated statistical uncertainty of 0.1 % for the energy loss of 12 MeV. The contribution from the noise can be neglected since the amplitude of noise is less than 2 mV and it is averaged over 40 pads and 20 samplings. The remaining factors for the worse resolution may be a reduction of the number of primary electrons contributing to the collected charge and any non-uniformity of the gain in one pad. Concerning the better resolution achieved at high gain, the collection efficiency of the primary electrons seems to be better at the high gain setting because the stronger electric field between the two THGEMs guides the electrons smoothly to the hole of next THGEM. There may also be effects from a relatively large amount of ion backflow as well as the related space charge in the reduction of the collection efficiency. The gain non-uniformity and the effect of ion backflow are as of yet inconclusive and still under evaluation.

The injection of a 10^6^ particles-per-pulse beam was successful for some time without any significant voltage variation. Eventually, discharge between the field cage and Si detector occurred. When the field cage discharged, there was no discharge of the DGTHGEMs. The discharge of field cage will hopefully be resolved by changing the position of the Si detectors as well as changing the field strength of the field cage. The physics experiment to measure the GMR in ^132^Xe will be performed in near future.
